# Supplementary material for: Robust differentiation of human enteroendocrine cells from intestinal stem cells
Source: Nat Commun. 2022 Jan 11;13:261. doi: 10.1038/s41467-021-27901-5 (PMC8752608; doi:10.1038/s41467-021-27901-5)
Supplement: Supplementary file 1 — Supplementary Information [file 41467_2021_27901_MOESM1_ESM.pdf]

Supplementary Figure 1

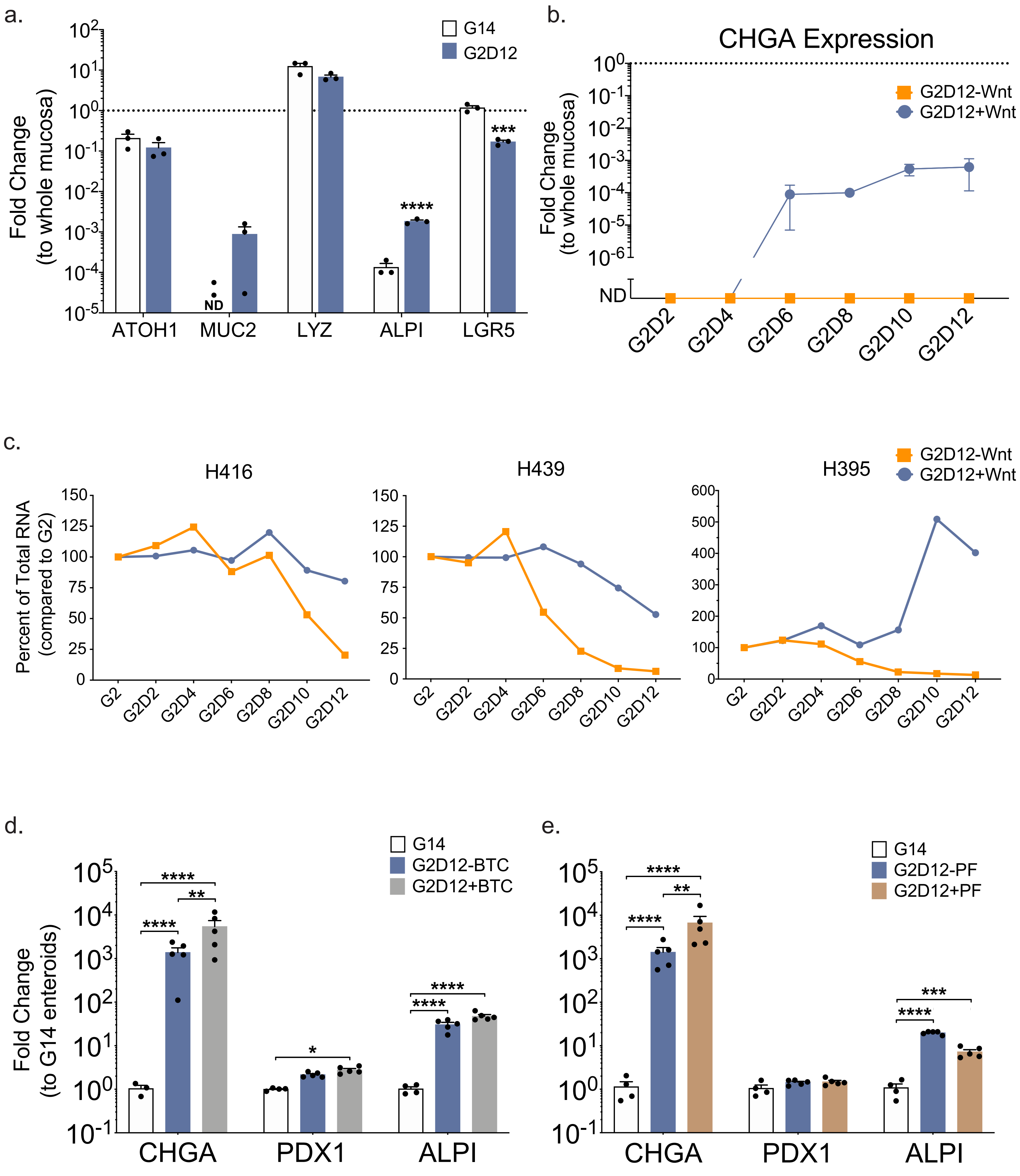

Supplementary Figure 1. Base Differentiation Media Components Important for Enteroendocrine Marker Expression

(a) qPCR analysis of intestinal lineage markers of enteroids grown in G14 or G2D12 compared to whole duodenal mucosa and normalized to *18S*. Dotted line denotes expression level in whole duodenal mucosa. Representative experiment showing n = 3 wells from each condition from a single enteroid line. *ATOH1* = atonal BHLH transcription factor 1, *MUC2* = mucin 2, *LYZ* = lysozyme, *ALPI* = intestinal alkaline phosphatase, *LGR5* = leucine-rich repeat-containing G-protein coupled receptor 5, ND = not detectable in one or more samples. ***p = 0.004; ****p < 0.0001.

(b) qPCR analysis of chromogranin A (*CHGA*) expression over time of enteroids grown in G2D12 with Wnt (G2D12+Wnt) or G2D12 without Wnt (G2D12-Wnt) compared to whole duodenal mucosa and normalized to *18S*. Dotted line denotes expression level in whole duodenal mucosa. Representative experiment showing n = 3 wells from each condition and timepoint from a single enteroid line. For G2D12+Wnt, only two wells expressed *CHGA* at G2D8 with the nondetectable sample being excluded from analysis. ND = not detectable.

(c) Time course study of total RNA levels from three enteroid lines grown in G2D12+Wnt or G2D12-Wnt, shown as a percent compared to RNA levels two days after starting experiment (G2). Representative experiment using an average of n = 3 wells from each condition and timepoint.

(d,e) qPCR analysis of intestinal lineage markers of enteroids grown in (**d**) G2D12 with betacellulin (G2D12+BTC) or G2D12 without betacellulin (G2D12-BTC) and in (**e**) G2D12 with PF06260933 (G2D12+PF) or G2D12 without PF06260933 (G2D12-PF) compared to G14 enteroids and normalized to *18S*. Representative experiments showing n = 3-5 wells from each condition from a single enteroid line. *PDX1* = pancreatic and duodenal homeobox 1. (**d**) *p = 0.0478; **p = 0.0023; ****p < 0.0001; (**e**) **p = 0.0080; ***p = 0.0003; ****p < 0.0001.

Bar and line (b) graphs show mean ± SEM; two-way ANOVA with Tukey correction for multiple comparisons (a,d,e). Each experiment repeated with at least three different enteroid lines.

Unless otherwise stated, specific conditions were excluded from statistical analysis if the data from one or more samples was labeled as not detectable. Source data are provided as a Source Data file.

Supplementary Figure 2

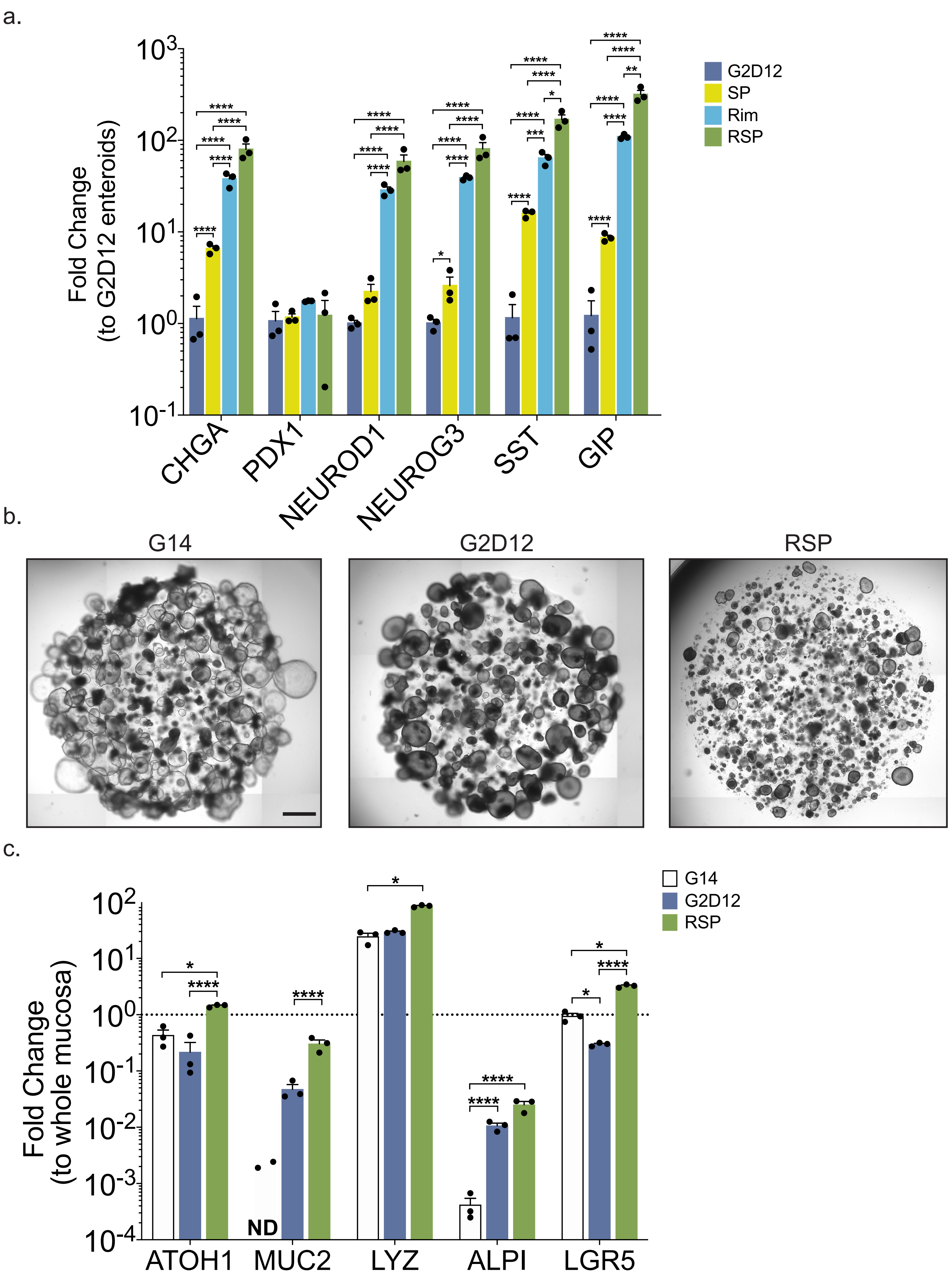

Supplementary Figure 2. Combination of Rimonabant and SP600125 Induces Enteroendocrine and Other Intestinal Lineage Markers

(a) qPCR analysis of enteroendocrine markers of enteroids grown in G2D12 with rimonabant (Rim), G2D12 with SP600125 (SP), and G2D12 with rimonabant and SP600125 (RSP) compared to enteroids grown in G2D12 and normalized to *18S*. Representative experiment showing n = 3 wells from each condition from single enteroid line. *CHGA* = chromogranin A, *PDX1* = pancreatic and duodenal homeobox 1, *NEUROD1* = neuronal differentiation 1, *NEUROG3* = neurogenin 3, *SST* = somatostatin, *GIP* = glucose-dependent insulinotropic peptide. *p = 0.0285 (*NEUROG3*), 0.0158 (*SST*); **p = 0.0074; ***p = 0.0003; ****p < 0.0001.

(b) Representative light microscopy of enteroids (whole well) grown in G14, G2D12, and RSP. Scale bar = 1mm.

(c) qPCR analysis of intestinal lineage markers of enteroids grown in G14, G2D12, and RSP compared to whole duodenal mucosa and normalized to *18S*. Dotted line denotes expression level in whole duodenal mucosa. Representative experiment showing n = 3 wells from each condition from a single enteroid line. *ATOH1* = atonal BHLH transcription factor 1, *MUC2* = mucin 2, *LYZ* = lysozyme, *ALPI* = intestinal alkaline phosphatase, *LGR5* = leucine-rich repeat-containing G-protein coupled receptor 5, ND = not detectable in one or more samples. *p = 0.0118 (*ATOH1*), 0.0108 (*LYZ*), 0.0334 (*LGR5*, G14 to G2D12), 0.0133 (*LGR5*, G14 to RSP); ****p < 0.0001.

Bars show mean ± SEM; two-way ANOVA with Tukey correction for multiple comparisons (a,c). Each experiment repeated with at least three different enteroid lines. Specific conditions were excluded from statistical analysis if the data from one or more samples was labeled as not detectable. Source data are provided as a Source Data file.

Supplementary Figure 3

a.

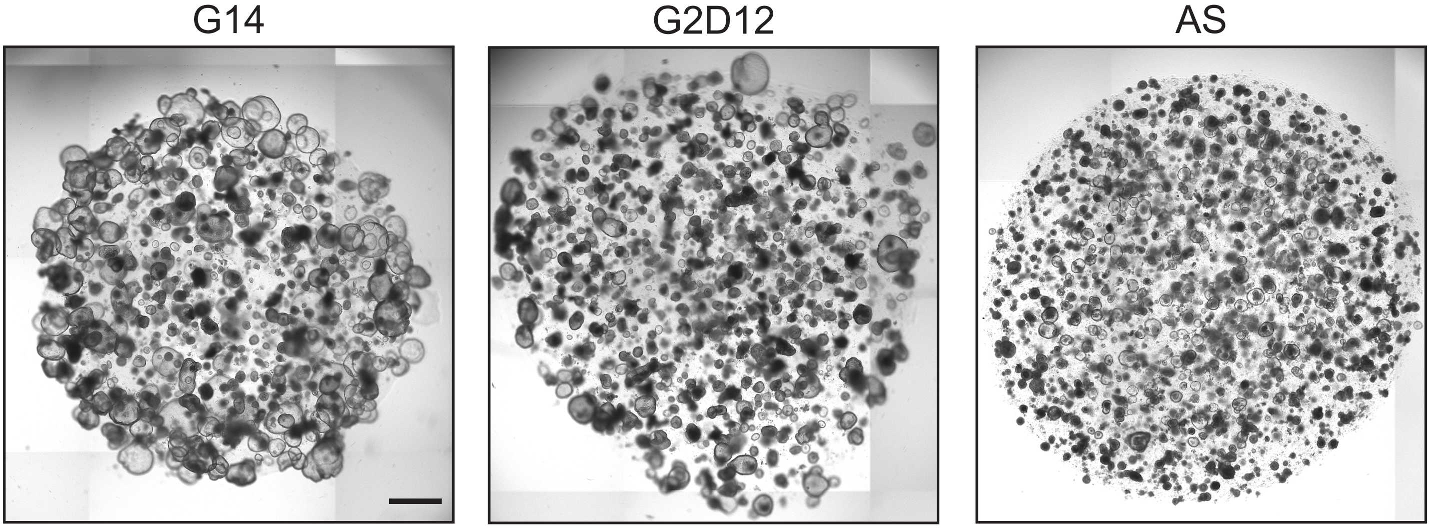

b.

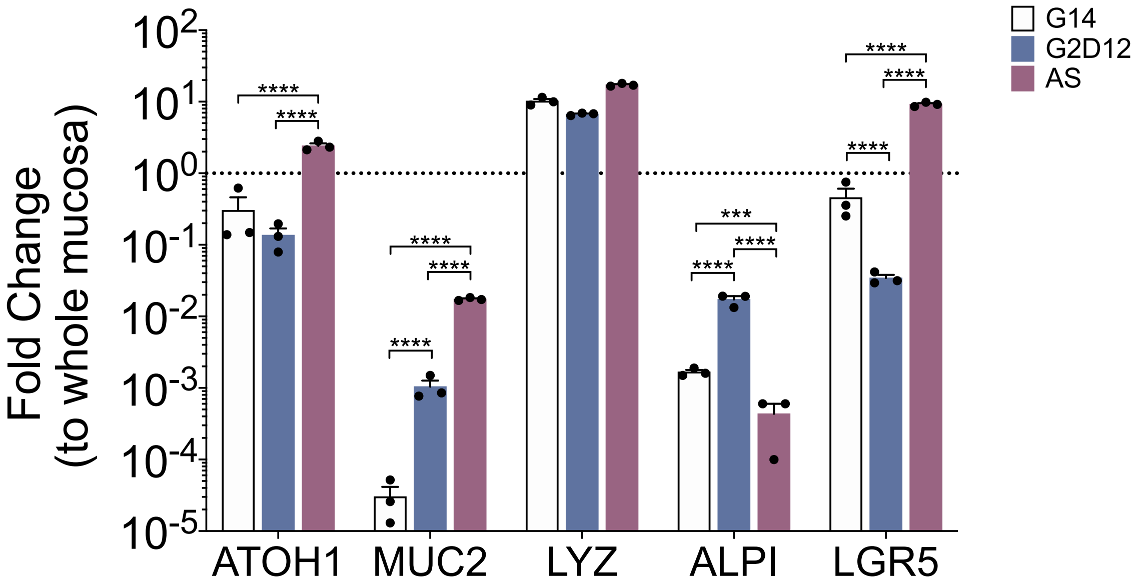

Supplementary Figure 3. AS1842856 Induces Specific Intestinal Lineage Markers

(a) Representative light microscopy of enteroids (whole well) grown in G14, G2D12, and G2D12 with AS1842856 (AS). Scale bar = 1mm.

(b) qPCR analysis of intestinal lineage markers of enteroids grown in G14, G2D12, and AS compared to whole duodenal mucosa and normalized to *18S*. Dotted line denotes expression level in whole duodenal mucosa. Representative experiment showing n = 3 wells from each condition from single enteroid line. *ATOH1* = atonal BHLH transcription factor 1, *MUC2* = mucin 2, *LYZ* = lysozyme, *ALPI* = intestinal alkaline phosphatase, *LGR5* = leucine-rich repeat-containing G-protein coupled receptor 5. ***p = 0.001, ****p < 0.0001.

Bars show mean ± SEM; two-way ANOVA with Tukey correction for multiple comparisons. Each experiment repeated with at least three different enteroid lines. Source data are provided as a Source Data file.

Supplementary Figure 4

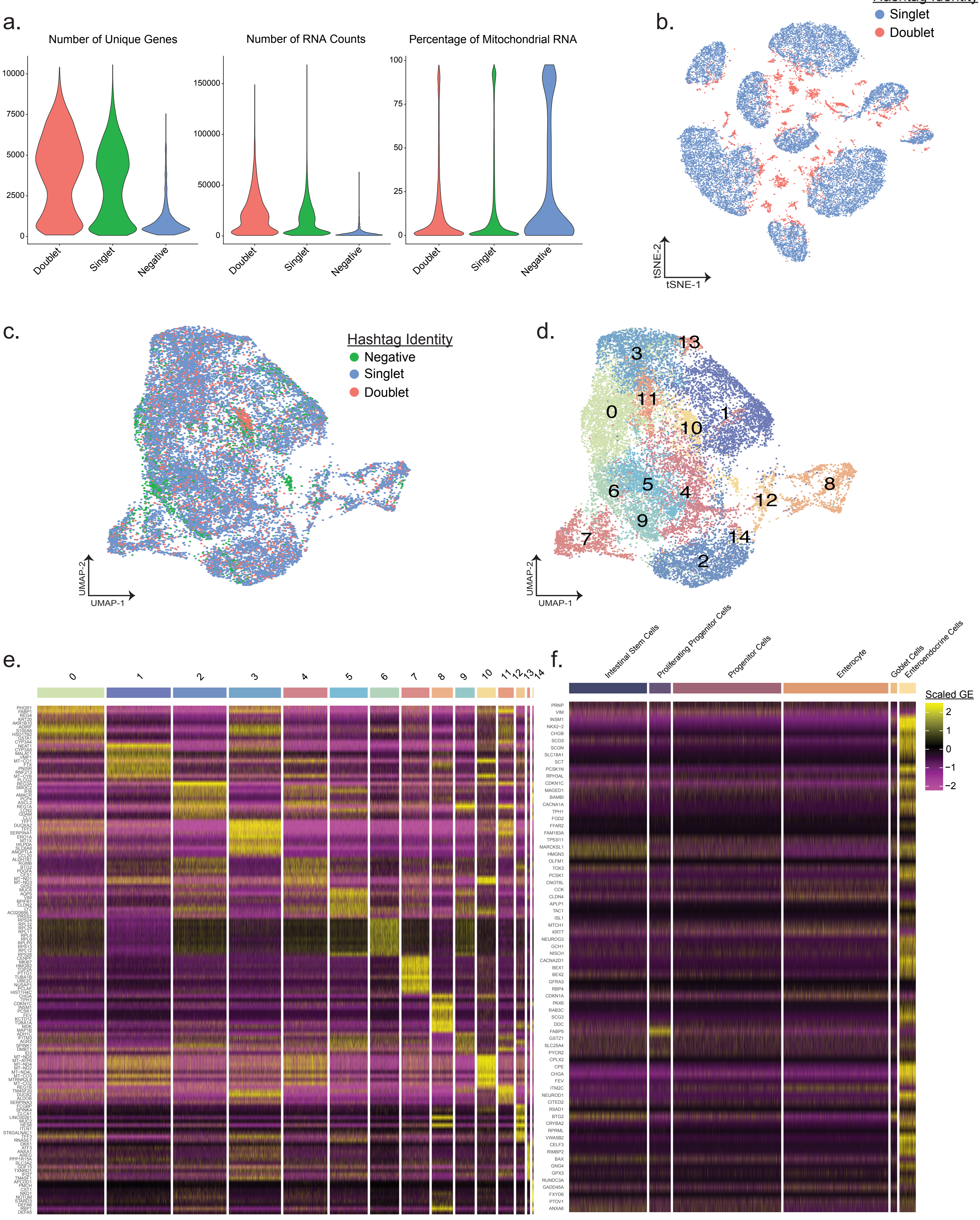

Supplementary Figure 4. Cellular Validation and Characterization of Enteroid Single Cell Transcriptomes

(a) Violin plots summarizing the distribution of genes (left), RNA counts or unique molecular identifiers (center), and the proportion of mitochondrial genes (right) within the scRNA-seq dataset.

(b) t-distributed stochastic neighbor embedding (tSNE) visualization of 23,335 cells from all samples with color denoting the hashtag identity. Singlet cells were denoted as having significant enrichment of only one hashtag signal per individual cell. Doublet cells had significant enrichment of more than one hashtag signal.

(c) Uniform manifold approximation and projection (UMAP) visualization of 25,673 cells originating from all samples, with color being used to distinguish between negative, doublet, or singlet cells.

(d) UMAP visualization from (c) with cells labeled by their Louvain cluster identity.

(e) Heatmap of the top 10 most differentially expressed genes per Louvain cluster compared to all other clusters. Differentially expressed genes were calculated using the Wilcoxon rank sum test and the top 10 genes of each cluster were selected based on their log fold enrichment in the specified cluster compared to all other clusters. Heatmap values corresponded to row-scaled Pearson residuals of gene expression that was normalized using regularized negative binomial regression.

(f) Heatmap of marker genes significantly enriched in *in vivo* EE cells isolated from the mouse small intestinal epithelial cell atlas. Heatmap values for display were calculated using similar methods seen in (e) and may not represent significantly enriched genes.

Supplementary Figure 5

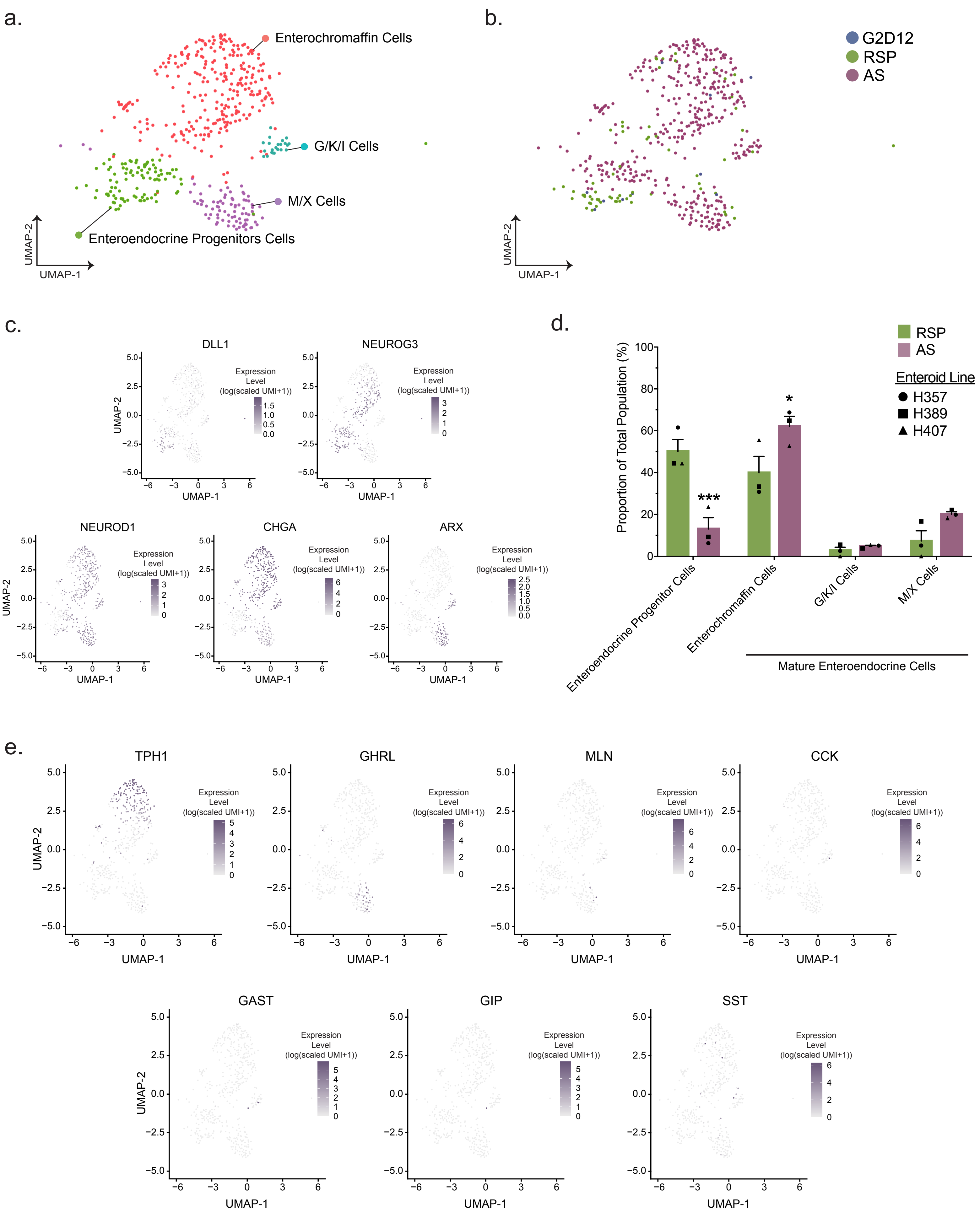

Supplementary Figure 5. Single Cell Analysis of Enteroendocrine Cells

(a) Uniform manifold approximation and projection (UMAP) visualization of 471 enteroendocrine cells from all samples and conditions, colored by cell identity.

(b) UMAP visualization from (a), colored by enteroid differentiation condition.

(c) Marker gene overlay for binned count-based expression level (log(scaled UMI + 1)) of various enteroendocrine marker genes, projected on the UMAP from (a). *DLL1* = delta like canonical notch ligand 1, *NEUROG3* = neurogenin 3, *NEUROD1* = neuronal differentiation 1, *CHGA* = chromogranin A, *ARX* = aristaless-related homeobox.

(d) Proportional abundance of enteroendocrine subsets by culture condition. G2D12 was not included due to low number of cells. Each culture condition consists of three different enteroid lines from distinct human donors, as denoted by data point shape. Bars show mean ± SEM; two-way ANOVA with Tukey correction for multiple comparisons. *p = 0.0160; ***p = 0.0002.

(e) Marker gene overlay for binned count-based expression level (log(scaled UMI + 1)) of various enteroendocrine hormone genes, projected on the UMAP from (a). *TPH1* = tryptophan hydroxylase 1, *GHRL* = ghrelin, *MLN* = motilin, *CCK* = cholecystokinin, *GAST* = gastrin, *GIP* = glucose-dependent insulinotropic peptide, *SST* = somatostatin. Source data are provided as a Source Data file.

Supplementary Figure 6

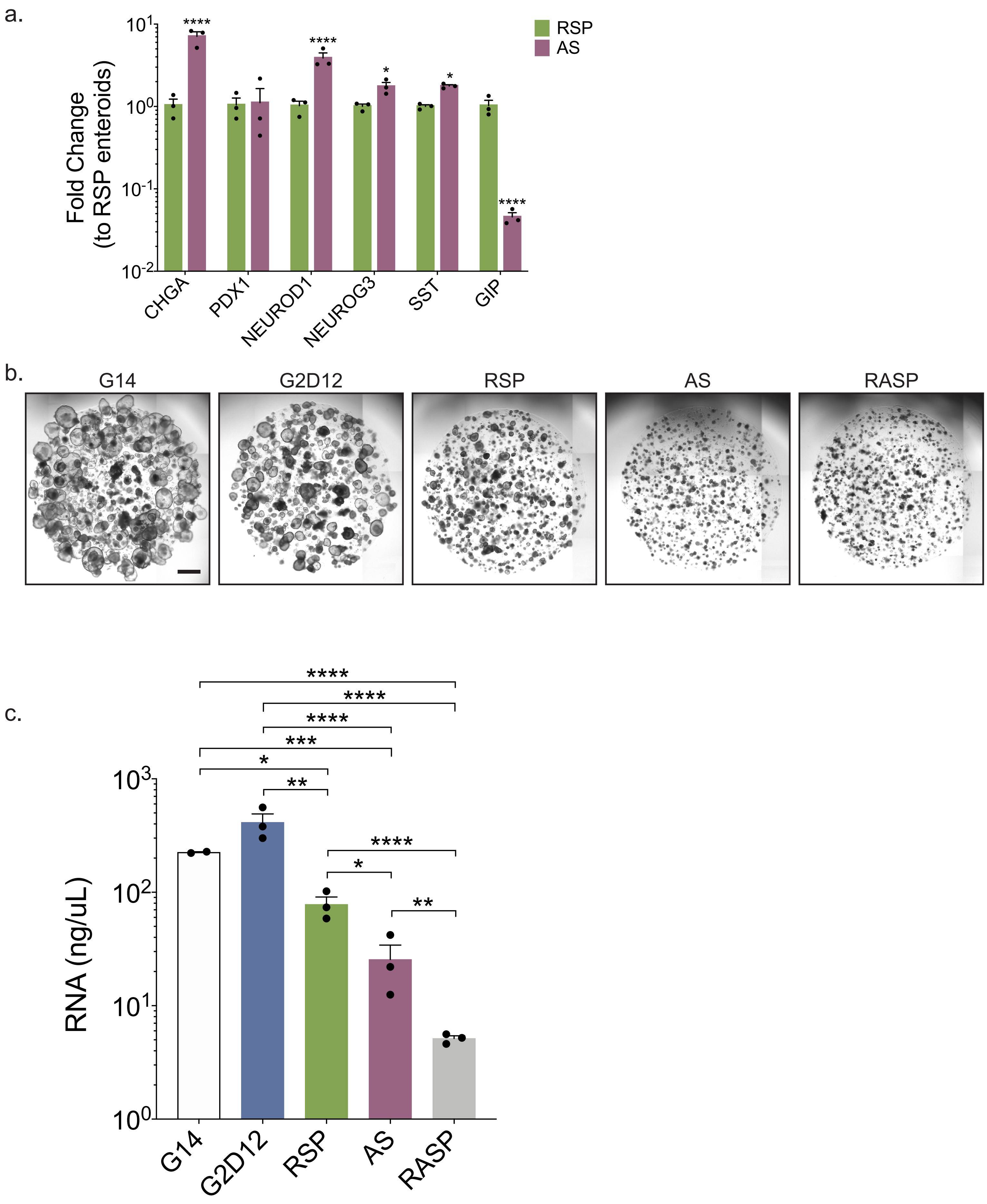

Supplementary Figure 6. Combination of AS1842856 and Rimonabant/SP600125 For All of Differentiation Leads to Reduction in Isolated RNA

(a) qPCR analysis of enteroendocrine markers of enteroids grown in AS compared to RSP and normalized to *18S*. Representative experiment showing n = 3 wells from each condition from a single enteroid line. *CHGA* = chromogranin A, *PDX1* = pancreatic and duodenal homeobox 1, *NEUROD1* = neuronal differentiation 1, *NEUROG3* = neurogenin 3, *SST* = somatostatin, *GIP* = glucose-dependent insulinotropic peptide. *p = 0.0407 (*NEUROG3*), 0.0306 (*SST*); ****p < 0.0001.

(b) Representative light microscopy of enteroids (whole well) grown in G14, G2D12, RSP, AS, and G2D12 with AS and RSP (RASP). Scale bar = 1 mm.

(c) Total RNA levels from enteroids grown in G14, G2D12, RSP, AS, and RASP. Representative results from n = 2-3 wells from each condition from a single enteroid line. *p = 0.0487 (G14 to RSP), 0.0142 (RSP to AS); **p = 0.0018 (G2D12 to RSP), 0.0039 (AS to RASP); ***p = 0.0004; ****p < 0.0001.

Bars show mean ± SEM; two-way ANOVA with Tukey correction for multiple comparisons (a); one-way ANOVA with Tukey correction for multiple comparisons (c). Each experiment repeated with at least three different enteroid lines. Source data are provided as a Source Data file.

Supplementary Figure 7

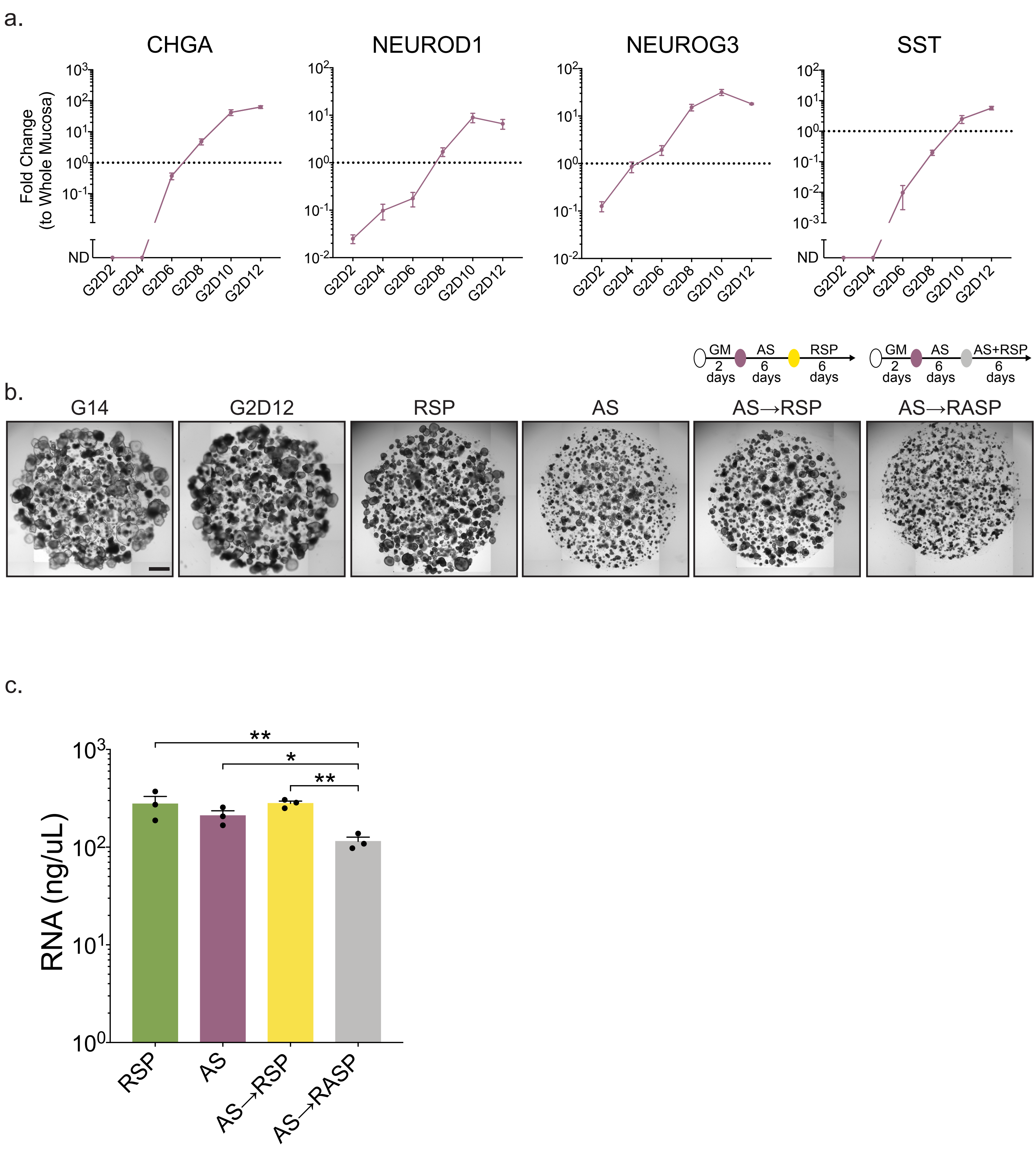

Supplementary Figure 7. Combination of AS1842856 and Rimonabant/SP600125 After Initial AS1842856 Exposure Yields Viable Enteroids

(a) qPCR analysis of enteroendocrine marker expression over time from enteroids grown in AS compared to whole duodenal mucosa and normalized to *18S*. RNA was collected every two days after start of differentiation. Dotted line denotes expression level in whole duodenal mucosa. Representative experiment showing n = 3 wells from each timepoint, except n = 2 for G2D12, from a single enteroid line. At G2D2, only two of three wells expressed *NEUROD1* and *NEUROG3*, with nondetectable samples excluded from analysis. *CHGA* = chromogranin A, *NEUROD1* = neuronal differentiation 1, *NEUROG3* = neurogenin 3, *SST* = somatostatin, ND = not detectable.

(b) Representative light microscopy of enteroids (whole well) grown in G14, G2D12, RSP, AS, AS→RSP, and AS→RASP. Specific culture schematics of AS→RSP and AS→RASP located above each panel, respectively. Scale bar = 1 mm.

(c) Total mRNA levels from enteroids grown in RSP, AS, AS→RSP, and AS→RASP. Representative experiment showing n = 3 wells from each condition from a single enteroid line. *p = 0.0456; **p = 0.0071 (RSP to AS→RASP), 0.0052 (AS→RSP to AS→RASP).

Bars and line graph show mean ± SEM; one-way ANOVA with Tukey correction for multiple comparisons (c). Each experiment repeated with at least three different enteroid lines. Source data are provided as a Source Data file.

Supplementary Figure 8

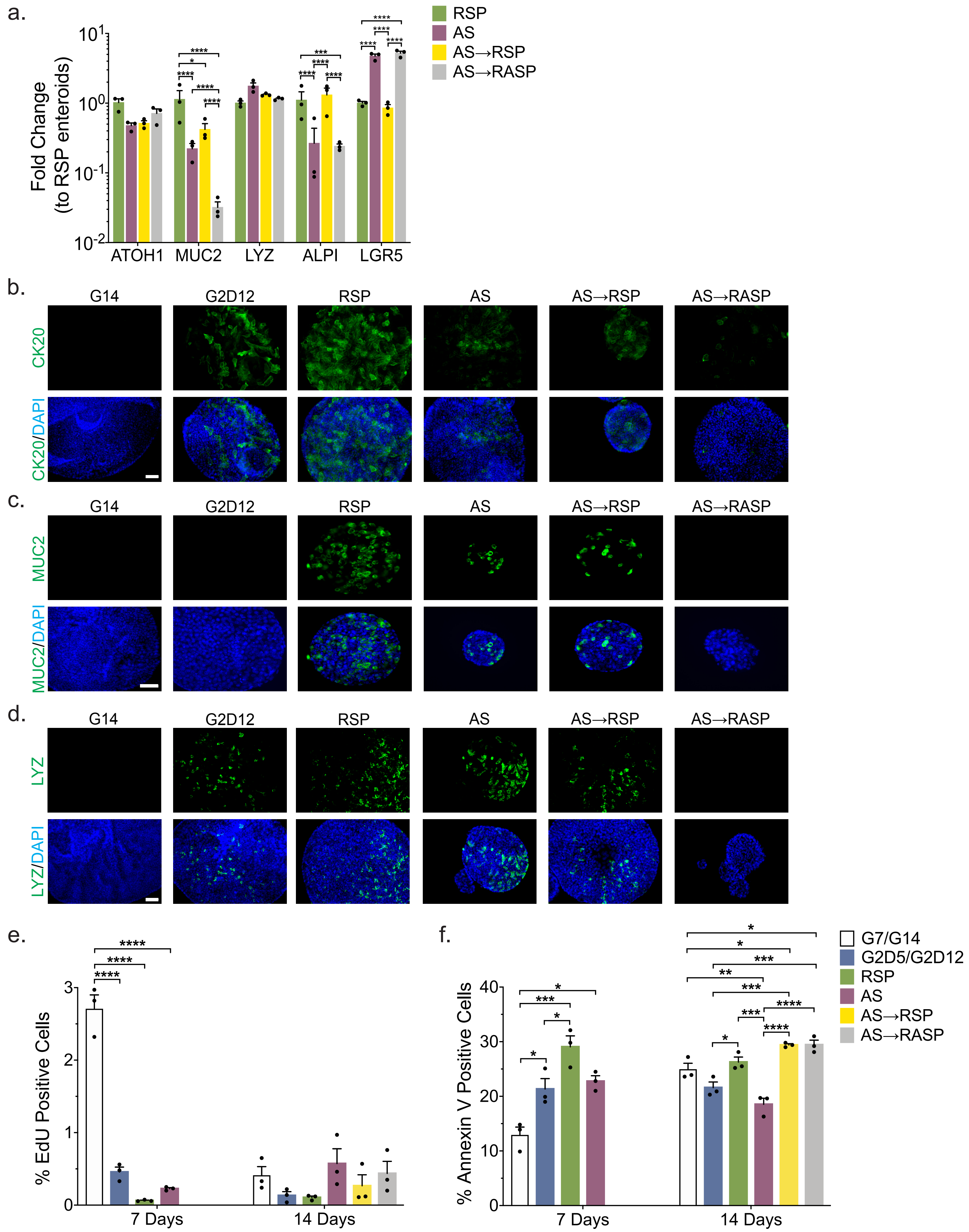

Supplementary Figure 8. Induction of Specific Intestinal Lineage Markers, Cell Viability, and Proliferation Compared Across Differentiation Methods

(a) qPCR analysis of intestinal lineage markers of enteroids grown in AS, AS→RSP, and AS→RASP compared to RSP and normalized to *18S*. Representative experiment showing n = 3 wells from each condition from single enteroid line. *ATOH1* = atonal BHLH transcription factor 1, *MUC2* = mucin 2, *LYZ* = lysozyme, ALPI = intestinal alkaline phosphatase, *LGR5* = leucine-rich repeat-containing G-protein coupled receptor 5. *p = 0.0266; ***p = 0.0002; ****p < 0.0001.

(b) Representative immunofluorescence staining of cytokeratin 20 (CK20, green) in enteroids treated with G14, G2D12, RSP, AS, AS→RSP, and AS→RASP. DNA (4′,6-diamidino-2-phenylindole (DAPI), blue). Scale bar = 50 µm.

(c) Representative immunofluorescence staining of mucin 2 (MUC2, green) in enteroids treated with G14, G2D12, RSP, AS, AS→RSP, and AS→RASP. DNA (4′,6-diamidino-2-phenylindole (DAPI), blue). Scale bar = 50 µm.

(d) Representative immunofluorescence staining of lysozyme (LYZ, green) in enteroids treated with G14, G2D12, RSP, AS, AS→RSP, and AS→RASP. DNA (4′,6-diamidino-2-phenylindole (DAPI), blue). Scale bar = 50 µm.

(e) Percentage of EdU-positive cells after a two-hour chase with 10 µM EdU in enteroids grown in their respective medias at either 7 days (left side) or 14 days (right side). ****p < 0.0001.

(f) Percentage of Annexin V-positive cells in enteroids grown in their respective medias at either 7 days (left side) or 14 days (right side). *p = 0.0276 (G7 to G2D5), 0.0121 (G7 to AS), 0.0428 (G2D5 to RSP), 0.0459 (G14 to AS→RSP), 0.0440 (G14 to AS→RASP), 0.0371 (G2D12 to RSP); **p = 0.0043; ***p = 0.0005 (G7 to RSP), 0.0008 (G2D12 to AS→RSP), 0.0007 (G2D12 to AS→RASP), 0.0008 (RSP to AS); ****p < 0.0001.

Bars and line graph show mean ± SEM; two-way ANOVA with Tukey correction for multiple comparisons (a); one-way ANOVA with Tukey correction for multiple comparisons (e,f) as

comparisons between 7 and 14 days were not made. Each experiment repeated with at least three different enteroid lines. Source data are provided as a Source Data file.

Supplementary Figure 9

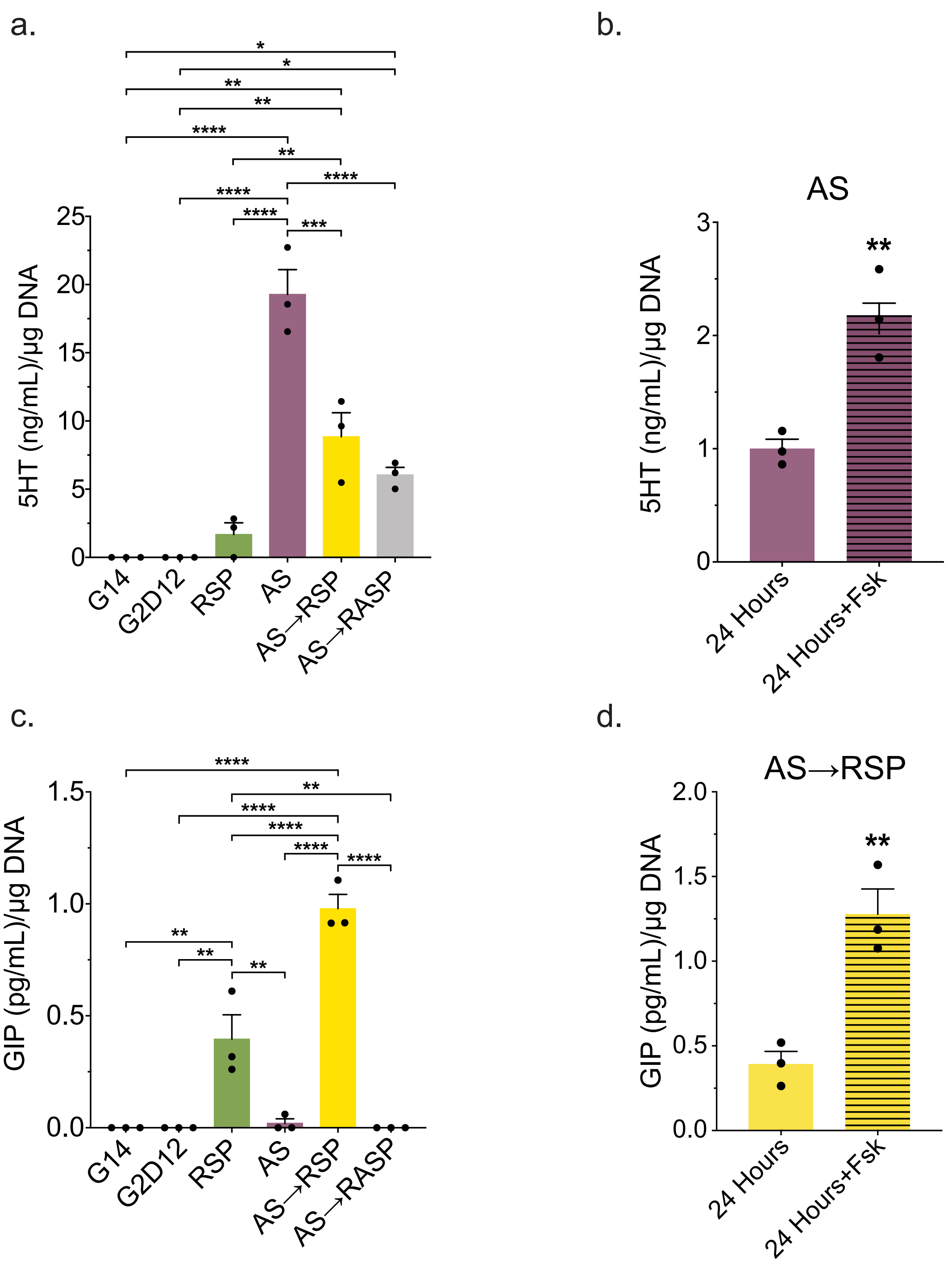

Supplementary Figure 9. Hormone Secretion of Different Conditions Controlled for Total DNA

(a) Serotonin (5HT) ELISA of conditioned media from the last two days of differentiation of enteroids grown in G14, G2D12, RSP, AS, AS→RSP, and AS→RASP controlled for total DNA from each sample. Representative experiment showing n = 3 wells from each condition from a single enteroid line. *p = 0.0223 (G14 to AS→RASP, G2D12 to AS→RASP); **p = 0.0012 (G14 to AS→RSP, G2D12 to AS→RSP), 0.0067 (RSP to AS→RSP); ***p = 0.0003, ****p < 0.0001.

(b) 5HT ELISA of AS conditioned media collected after 24 hours on day 13 (solid bar) and after 24 hours with forskolin (Fsk) on day 14 (striped bar) controlled for total DNA from each sample. Representative experiment showing n = 3 wells from each condition from a single enteroid line. **p = 0.0081.

(c) Glucose-dependent insulinotropic peptide (GIP) ELISA of conditioned media from the last two days of differentiation of enteroids grown in G14, G2D12, RSP, AS, AS→RSP, and AS→RASP controlled for total DNA from each sample. Representative experiment showing n = 3 wells from each condition from a single enteroid line. **p = 0.0017 (G14 to RSP, G2D12 to RSP, RSP to AS→RASP), 0.0027 (AS to RSP); ****p < 0.0001.

(d) GIP ELISA of AS→RSP conditioned media collected after 24 hours on day 13 (solid bar) and after 24 hours with Fsk on day 14 (striped bar) controlled for total DNA from each sample. Representative experiment showing n = 3 wells from each condition from a single enteroid line. **p = 0.0061.

Bars show mean ± SEM; one-way ANOVA with Tukey correction for multiple comparisons (a,c); two-tailed unpaired *t* test (b,d). To control for enteroid number, protein concentrations were divided by DNA concentration. Each experiment repeated with at least three different enteroid lines. Source data are provided as a Source Data file.

Supplementary Figure 10

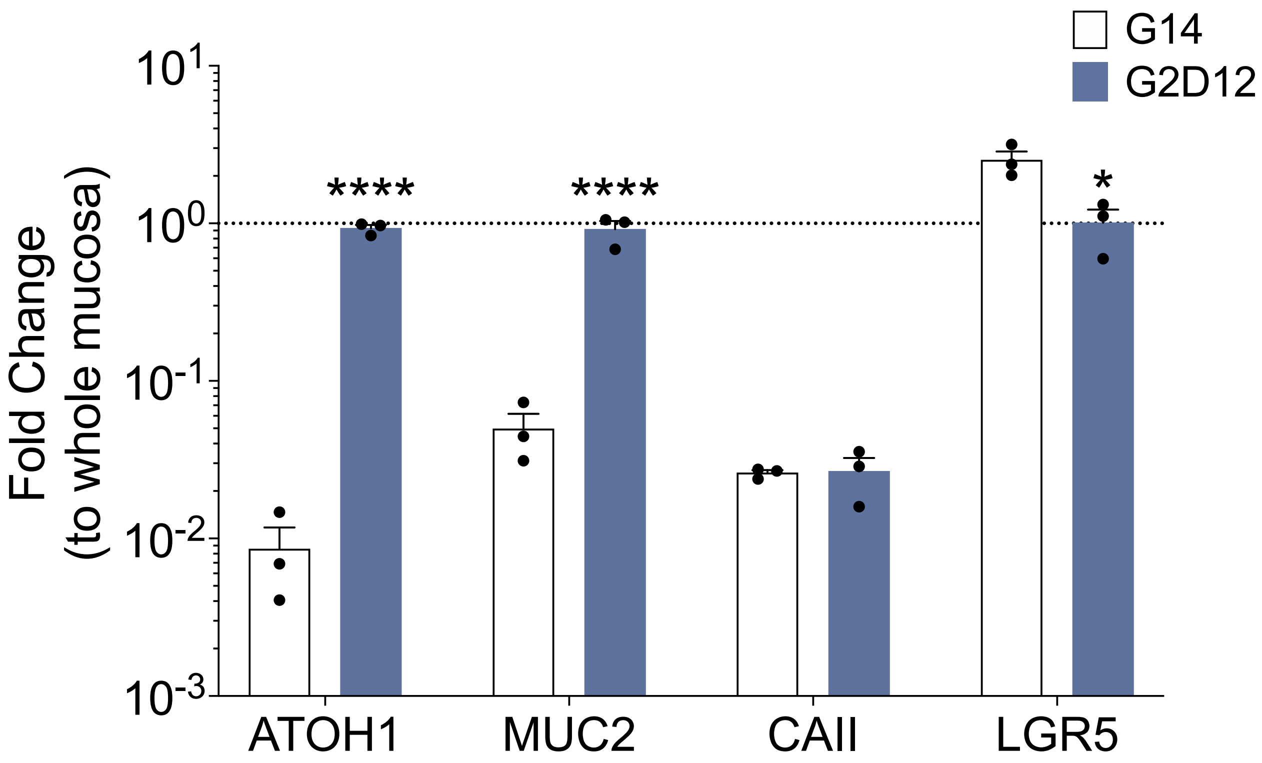

Supplementary Figure 10. Induction of Other Lineage Markers in Rectoids

qPCR analysis of intestinal lineage markers of rectoids grown in G14 and G2D12 compared to whole rectal mucosa and normalized to *18S*. Dotted line denotes expression level in whole rectal mucosa. Representative experiment showing n = 3 wells from each condition from a single rectoid line. *ATOH1* = atonal BHLH transcription factor 1, *MUC2* = mucin 2, *CAII* = carbonic anhydrase II, *LGR5* = leucine-rich repeat-containing G-protein coupled receptor 5. *p = 0.0150; ****p < 0.0001.

Bars show mean ± SEM; two-way ANOVA with Tukey correction for multiple comparisons. Experiment repeated with at least three different rectoid lines. Source data are provided as a Source Data file.

Supplementary Figure 11

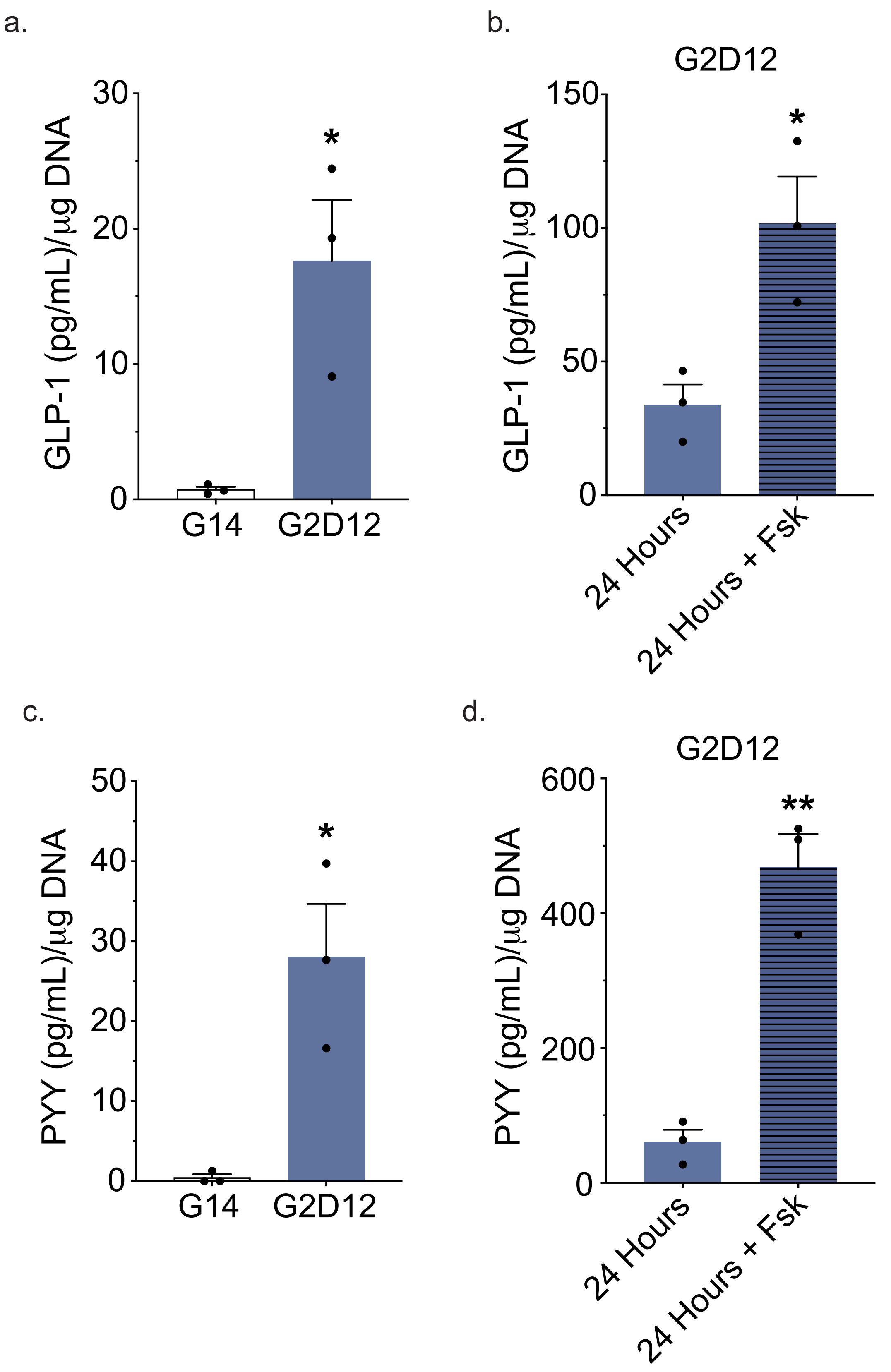

Supplementary Figure 11. Rectoid Hormone Secretion Controlled for Total DNA

(a) Glucagon-like peptide-1 (GLP-1) ELISA of conditioned media from the last two days of differentiation of rectoids grown in G14 and G2D12 controlled for total DNA from each sample. Representative experiment showing n = 3 wells from each condition from a single rectoid line. *p = 0.0202.

(b) GLP-1 ELISA of G2D12 conditioned media collected after 24 hours on day 13 (solid bar) and after 24 hours with forskolin (Fsk) on day 14 (striped bar) controlled for total DNA from each sample. Representative experiment showing n = 3 wells from each condition from a single rectoid line. *p = 0.0233.

(c) Peptide YY (PYY) ELISA of conditioned media from the last two days of differentiation of rectoids grown in G14 and G2D12 controlled for total DNA from each sample. Representative experiment showing n = 3 wells from each condition from a single rectoid line. *p = 0.0145.

(d) PYY ELISA of G2D12 conditioned media collected after 24 hours on day 13 (solid bar) and after 24 hours with Fsk on day 14 (striped bar) controlled for total DNA from each sample. Representative experiment showing n = 3 wells from each condition from a single rectoid line. **p = 0.0016.

Bars show mean ± SEM; two-tailed unpaired *t* test (a,b,c,d). To control for rectoid number, protein concentrations were divided by DNA concentration. Each experiment repeated with at least three different rectoid lines. Source data are provided as a Source Data file.

Supplementary Figure 12

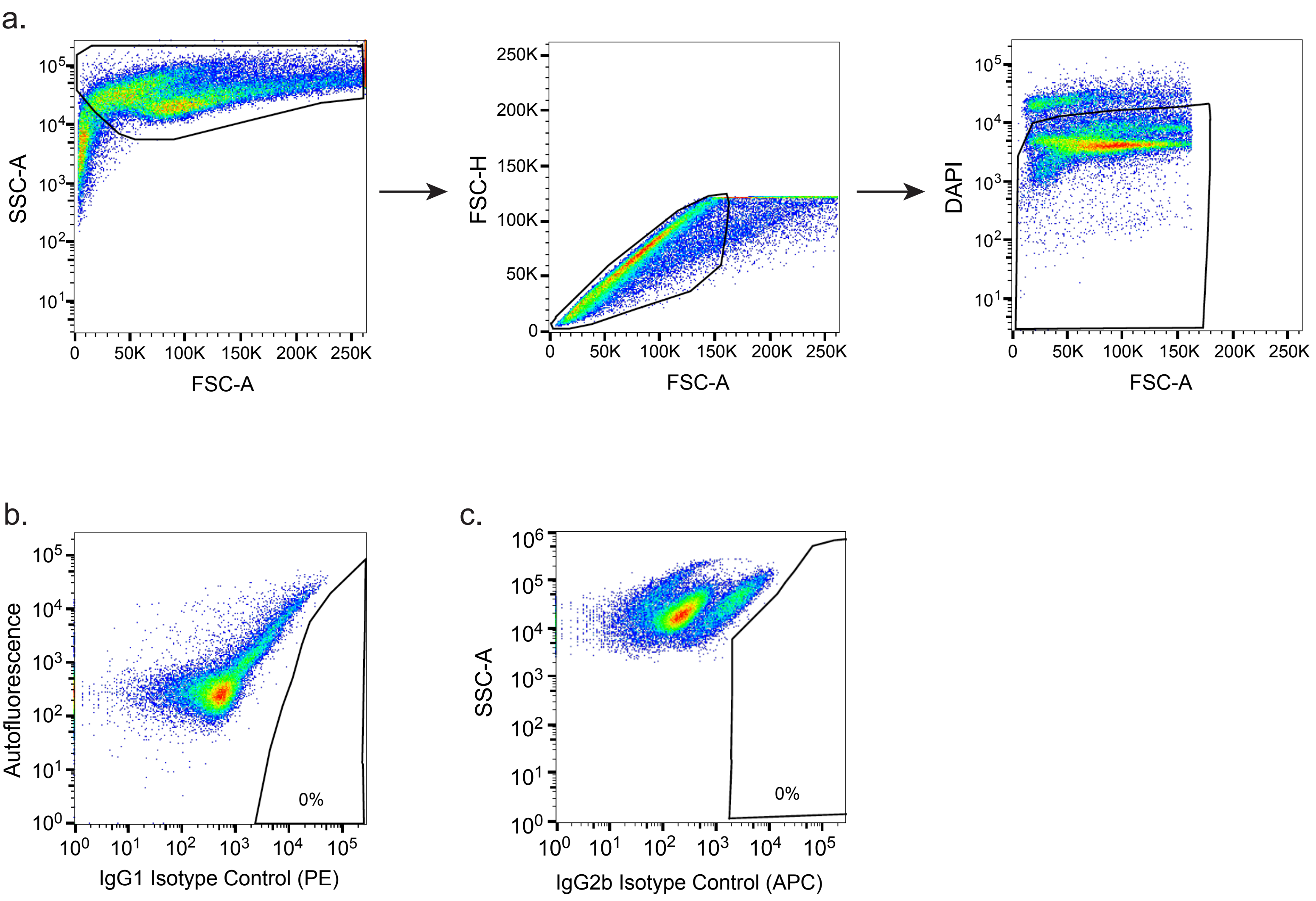

Supplementary Figure 12. Gating Strategy for Flow Cytometry

(a) Cells are differentiated from cellular debris based on their forward and side scatter area (FSC-A and SSC-A, respectively) parameters. Cells are then examined based on their FSC-A and FSC-Height (H) to exclude doublets. 4′,6-diamidino-2-phenylindole (DAPI) staining is then utilized to identify dead cells, with DAPI high-positive cells being excluded from further gating.

(b) The CHGA-positive gate for enteroids is set by using an IgG1 K isotype control conjugate with phycoerythrin (PE).

(c) The CHGA-positive gate for rectoids is set by using an IgG2b K isotype control conjugate with Alexa fluor 647 (APC).

Supplementary Table 1. Growth and Differentiation Media Components

ENTEROID		RECTOID	
**Growth Media**	**Differentiation Media**	**Growth Media**	**Differentiation Media**
L-WRN conditioned media (50% v/v)		L-WRN conditioned media (65% v/v)	
DMEM/F12 (45% v/v)		DMEM/F12 (30% v/v)	
Glutamax (1% v/v)		Glutamax (1% v/v)	
N-2 Supplement (1% v/v)		N-2 Supplement (1% v/v)	
B-27 Supplement (1% v/v)		B-27 Supplement (1% v/v)	
HEPES (10mM)		HEPES (10mM)	
Primocin (100μg/mL)		Primocin (100μg/mL)	
Normocin (100μg/mL)		Normocin (100μg/mL)	
A83-01 (500nM)		A83-01 (500nM)	
N-Acetyl-cysteine (500μM)		N-Acetyl-cysteine (500μM)	
Recombinant Murine EGF (50ng/mL)		Recombinant Murine EGF (50ng/mL)	
Human [Leu15] Gastrin I (50nM)		Human [Leu15] Gastrin I (50nM)	
Nicotinamide (10mM)	DAPT (20μM)	Nicotinamide (10mM)	DAPT (20μM)
SB202190 (10μM)	Betacellulin (20ng/mL)	SB202190 (10μM)	Betacellulin (20ng/mL)
	Tubastatin-A (10μM)	Prostaglandin-E2 (10nM)	Tubastatin-A (10μM)
	PF06260933 (6μM)		PF06260933 (6μM)
	Tranylcypromine (1.5μM)		Tranylcypromine (1.5μM)
	Rimonabant (10μM)		
	SP600125 (10μM)		
	AS1842856 (100nM)		

Supplementary Table 1. Growth and Differentiation Media Components

Growth factors, supplements, and small molecules common to both growth and differentiation medias are listed first. The L-WRN cell line was used to produce conditioned media containing Wnt3a, Noggin and R-spondin 3. The HA-R-Spondin1-Fc 293T cell line was used to produce conditioned media with only R-spondin 1. This conditioned media, with supplemented Noggin (100 ng/mL), was used to make Wnt3a-free differentiation media.

Supplementary Table 2. Description of Samples

**Application**	**Age Range (years)**	**Number of Males**	**Number of Females**
Enteroid Lines	13-21	4	4
Rectoid Lines	12-18	2	6
Duodenal mucosa RNA	55-82	1	2
Rectal mucosa RNA	15-18	0	3

Supplementary Table 2. Description of Samples

Samples used to produce organoid cell lines are labeled as either enteroid or rectoid lines, while those used as a duodenal or rectal whole mucosa controls are labeled as mucosa RNA. For each application, the age range and number of males and females are noted. All biopsies and resections were determined to be healthy, and from patients without known gastrointestinal disease, before being provided to the researchers de-identified, with age and sex not known until after completion of experiments.

Supplementary Table 3. Taqman qPCR Primers

**Name**	**Abbreviation**	**Identifier**
18S	*18S*	Hs99999901_s1
Intestinal alkaline phosphatase	*ALPI*	Hs00357579_g1
Atonal bHLH transcription factor 1	*ATOH1*	Hs00944192_s1
Carbonic anhydrase II	*CAII*	Hs01070108_m1
Cholecystokinin	*CCK*	Hs00174937_m1
Chromogranin A	*CHGA*	Hs00900370_m1
GATA binding protein 4	*GATA4*	Hs00171403_m1
Glucose-dependent insulinotropic polypeptide	*GIP*	Hs00175030_m1
Glucagon	*GCG*	Hs01031536_m1
Leucine-rich repeat-containing G-protein coupled receptor 5	*LGR5*	Hs00969422_m1
Lysozyme	*LYZ*	Hs00426232_m1
Mucin 2	*MUC2*	Hs03005103_g1
Neuronal differentiation 1	*NEUROD1*	Hs01922995_s1
Neurogenin 3	*NEUROG3*	Hs01875204_s1
Paired box 4	*PAX4*	Hs00173014_m1
Pancreatic and duodenal homeobox 1	*PDX1*	Hs00236830_m1
Peptide YY	*PYY*	Hs00373890_g1
Somatostatin	*SST*	Hs00356144_m1

Supplementary Table 4. Reagents and Resources

REAGENT or RESOURCE	SOURCE	CATALOG NUMBER
**Primary Antibodies (Dilution used)**		
Alexa Fluor 647-Conjugated Anti-Chromogranin A Antibody (1:100)	Novus Biologicals	NBP2-47850AF647
Alexa Fluor 647-Conjugated Mouse IgG2b kappa Isotype Control Antibody (1:100)	Biolegend	400330
Anti-Chromogranin A Antibody (1:100)	Agilent/Dako	M086901-2
Anti-Chromogranin A Antibody (1:100)	Millipore Sigma	HPA017369-100UL
Anti-Cholecystokinin Antibody (1:100)	Abcam	Ab27441
Anti-Cytokeratin 20 Antibody (1:50)	Thermo Fisher Scientific	17329-1-AP
Anti-GIP Antibody (1:100)	Invitrogen	PA5-76867
Anti-GLP-1 Antibody (1:100)	Abcam	Ab23468
Anti-Lysozyme Antibody (1:50)	Novus Biologicals	NB100-63062
Anti-MUC2 Antibody (1:50)	Novus Biologicals	NBP1-31231
Anti-PYY Antibody (1:50)	Mybiosource	MBS9208739
Anti-Serotonin Antibody (1:100)	Abcam	ab66047
Anti-Somatostatin Antibody (1:100)	R&D Systems	mab2358
APC anti-human β2-microglobulin Antibody (1:25)	Biolegend	316311
APC anti-human CD298 Antibody (1:25)	Biolegend	341706
PE-conjugated Anti-Chromogranin antibody (1:100)	BD Biosciences	564563
PE-conjugated Mouse IgG1 kappa Isotype Control Antibody (1:200)	BD Biosciences	554680
TotalSeq™-B0251 anti-human Hashtag 1 Antibody (1:25)	Biolegend	394631
TotalSeq™-B0252 anti-human Hashtag 2 Antibody (1:25)	Biolegend	394633
TotalSeq™-B0253 anti-human Hashtag 3 Antibody (1:25)	Biolegend	394635
TotalSeq™-B0254 anti-human Hashtag 4 Antibody (1:25)	Biolegend	394637
TotalSeq™-B0255 anti-human Hashtag 5 Antibody (1:25)	Biolegend	394639
TotalSeq™-B0256 anti-human Hashtag 6 Antibody (1:25)	Biolegend	394641
TotalSeq™-B0257 anti-human Hashtag 7 Antibody (1:25)	Biolegend	394643
TotalSeq™-B0258 anti-human Hashtag 8 Antibody (1:25)	Biolegend	394645
TotalSeq™-B0259 anti-human Hashtag 9 Antibody (1:25)	Biolegend	394647
**Secondary Antibodies**		
Donkey anti-Goat IgG (H+L) Cross-Adsorbed Secondary Antibody, Alexa Fluor 488 (1:400)	Invitrogen	A-11055
Donkey anti-Mouse IgG (H+L) Cross-Adsorbed Secondary Antibody, Alexa Fluor 488 (1:400)	Invitrogen	A-21202
Donkey anti-Mouse IgG (H+L) Cross-Adsorbed Secondary Antibody, Alexa Fluor 647 (1:400)	Invitrogen	A-31571
Donkey anti-Rabbit IgG (H+L) Cross-Adsorbed Secondary Antibody, Alexa Fluor 647 (1:400)	Invitrogen	A-31573
Donkey anti-Rat IgG (H+L) Cross-Adsorbed Secondary Antibody, Alexa Fluor 488 (1:400)	Invitrogen	A-21208
**Chemicals and Enzymes**		

4′,6-diamidino-2-phenylindole (DAPI)	Thermo Fisher Scientific	D1306
A-8301	Millipore Sigma	SML0788
AS1842856	Millipore Sigma	344355
B-27 Supplement	Thermo Fisher Scientific	12587010
Betacellulin	Peprotech	100-50
Bovine serum albumin	Millipore Sigma	05470-1G
Cell Recovery Solution	Corning	354253
Cell Staining Buffer	Biolegend	420201
Collagenase Type I	Thermo Fisher Scientific	17018029
DAPT	Selleckchem	S2215
Diprotin A	Tocris	6019
Advanced DMEM/F12	Thermo Fisher Scientific	12634028
DMEM Ca2+ Free	Thermo Fisher Scientific	21068-028
RNAse Inhibitor	Thermo Fisher Scientific	N8080119
EGF, Recombinant Murine	Peprotech	315-09
Fetal Bovine Serum Certified USA Origin	Thermo Fisher Scientific	16000044
Forskolin	Tocris	1099
Gastrin I [Leu15], Human	Millipore Sigma	G9145
GlutaMAX	Thermo Fisher Scientific	35050061
HEPES	Thermo Fisher Scientific	15630080
Matrigel, growth factor reduced, phenol red-free	Corning	356231
N-2 Supplement	Thermo Fisher Scientific	17502001
N-Acetyl-cysteine	Millipore Sigma	A7250
Nicotinamide	Millipore Sigma	N0636
Noggin, Recombinant Murine	Peprotech	250-38
Normocin	Invivogen	ant-nr-2
Paraformaldehyde, 32%	Electron Microscopy Sciences (VWR)	15714-S
PF-06260933	Millipore Sigma	PZ0272
Primocin	Invivogen	ant-pm-2
Prolong Gold Antifade Mountant	Thermo Fisher Scientific	P36930
Prostaglandin-E2	Millipore Sigma	P0409
Rimonabant	Millipore Sigma	SML0800
SB202190	Millipore Sigma	S7067
SP600125	Millipore Sigma	S5567
Tranylcypromine	Tocris	3852
TRI Reagent®	Millipore Sigma	T9424-200ML
Triton X-100	Millipore Sigma	T8787-50ML
TruStain FcX™ (Fc Receptor Blocking Solution), Human	Biolegend	422301
TrypLE Express	Thermo Fisher Scientific	12605-010
Tubastatin-A	Selleckchem	S8049
Y-27632 dihydrochloride	Tocris	1254
**Commercial Assays**		
3’ Feature Barcode Kit, 16rxn Dual Indexing Compatible	10X Genomics	1000269

Click-IT EdU Alexa Fluor 488 Flow Cytometry Assay Kit	Thermo Fisher Scientific	C10420
Chromium NextGem Chip G Single Cell Kit	10X Genomics	1000127
Chromium Next Gem Single Cell 3' GEM, Library and Gel Bead Kit v3.1, 4rxn Dual Index Compatible	10X Genomics	1000269
Dead Cell Apoptosis Kit with Annexin V Alexa Fluor 488 & Propidium Iodide	Thermo Fisher Scientific	V13241
Dead Cell Removal Kit	Miltenyi Biotec	130-090-101
Direct-zol RNA Microprep Kit	Zymo Research	R2061
High-Capacity cDNA Reverse Transcription Kit	Thermo Fisher Scientific	4368813
Human GIP (total) ELISA Kit	Millipore Sigma	EZHGIP-54K
Human GLP-1 (7-36) ELISA Kit	Abcam	Ab184857
Human PYY ELISA Kit	Abcam	Ab255727
Human Serotonin ELISA Assay Kit	Eagle Biosciences/DLD Diagnostika GmbH	SER39-K01
TaqMan™ Universal PCR Master Mix	Thermo Fisher Scientific	4304437
