# Supplementary material for: Robust differentiation of human enteroendocrine cells from intestinal stem cells
Source: Nat Commun. 2022 Jan 11;13:261. doi: 10.1038/s41467-021-27901-5 (PMC8752608; doi:10.1038/s41467-021-27901-5)
Supplement: Supplementary file 3 — Reporting Summary [file 41467_2021_27901_MOESM3_ESM.pdf]

nature research

Corresponding author(s): David Breault, MD, PhD

Last updated by author(s): 12/1/2021

Reporting Summary

Nature Research wishes to improve the reproducibility of the work that we publish. This form provides structure for consistency and transparency in reporting. For further information on Nature Research policies, see our Editorial Policies and the Editorial Policy Checklist.

Statistics

For all statistical analyses, confirm that the following items are present in the figure legend, table legend, main text, or Methods section.

n/a	Confirmed	
☐	☒	The exact sample size (*n*) for each experimental group/condition, given as a discrete number and unit of measurement
☐	☒	A statement on whether measurements were taken from distinct samples or whether the same sample was measured repeatedly
☐	☒	The statistical test(s) used AND whether they are one- or two-sided<br>*Only common tests should be described solely by name; describe more complex techniques in the Methods section.*
☒	☐	A description of all covariates tested
☐	☒	A description of any assumptions or corrections, such as tests of normality and adjustment for multiple comparisons
☐	☒	A full description of the statistical parameters including central tendency (e.g. means) or other basic estimates (e.g. regression coefficient) AND variation (e.g. standard deviation) or associated estimates of uncertainty (e.g. confidence intervals)
☐	☒	For null hypothesis testing, the test statistic (e.g. *F*, *t*, *r*) with confidence intervals, effect sizes, degrees of freedom and *P* value noted<br>*Give P values as exact values whenever suitable.*
☒	☐	For Bayesian analysis, information on the choice of priors and Markov chain Monte Carlo settings
☒	☐	For hierarchical and complex designs, identification of the appropriate level for tests and full reporting of outcomes
☐	☒	Estimates of effect sizes (e.g. Cohen's *d*, Pearson's *r*), indicating how they were calculated

Our web collection on statistics for biologists contains articles on many of the points above.

Software and code

Policy information about availability of computer code

Data collection	EVOS FL Auto 2 imaging software (revision 2.0.1732.0) and Nikon Elements (NIS-Elements AR Ver5.21.00) were used to acquire images.
Data analysis	Fiji (v2.10/1.53c) was used to analyze immunoflourescence data as described in the Methods section<br>BD FACSDiva v8.02 was used to obtain flow cytometry data<br>FlowJo v10.6.2 was used to analyze flow cytometry data<br>Graphpad Prism 8 was used to perform statistical analysis on all numerical data<br>Cellranger 5.0.1 was used to demultiplex and align the raw sequencing reads<br>R v4.0.3 was used to analyze scRNAseq data within Rstudio with the following packages:<br>Seurat 4.0.1, tidyverse 1.3.1, PNWColors 0.1.0, scTransform 0.3.2, cowplot 1.1.1<br>RNA velocity was calculated using Python v3.7.10 and the following packages:<br>scVelo 0.2.3, Scanpy 1.7.1, Velocyto 0.17.15

For manuscripts utilizing custom algorithms or software that are central to the research but not yet described in published literature, software must be made available to editors and reviewers. We strongly encourage code deposition in a community repository (e.g. GitHub). See the Nature Research guidelines for submitting code & software for further information.

Data

Policy information about availability of data

All manuscripts must include a data availability statement. This statement should provide the following information, where applicable:

- Accession codes, unique identifiers, or web links for publicly available datasets
- A list of figures that have associated raw data
- A description of any restrictions on data availability

Data generated in this study have been deposited in Gene Expression Omnibus with the accession code GSE178342. For access, please visit https://www.ncbi.nlm.nih.gov/geo/query/acc.cgi?acc=GSE178342. All other relevant data supporting the key findings of this study are available within the article and its Supplementary Information files. Source data are provided with this paper.

Field-specific reporting

Please select the one below that is the best fit for your research. If you are not sure, read the appropriate sections before making your selection.

- [x] Life sciences
- [ ] Behavioural & social sciences
- [ ] Ecological, evolutionary & environmental sciences

For a reference copy of the document with all sections, see nature.com/documents/nr-reporting-summary-flat.pdf

Life sciences study design

All studies must disclose on these points even when the disclosure is negative.

Sample size	For all experiments, n = 3 was chosen as the minimal replicate number based on prior studies showing significance with similar sample sizes. We determined this to be sufficient due to relatively low variability between samples in the same group, allowing for clear delineation of differences between groups and statistical significance. The only exception to this is Supplementary Figure 4c, as the G14 group only had an n = 2 due to the loss of a sample. However, repeats of this study yielded similar results and the n = 2 still allowed for statistical significance, so we included it in this study.
Data exclusions	Data was not excluded from analysis.
Replication	All replication attempts from the same organoid line were successful and showed results similar to those in the paper. Due to the variability seen between human samples, each experiment also was performed on at least three separate human organoid lines. These findings also showed similar results to the results shown in the paper, as evidenced by Figures 2d, 3d, 4c, 5b, 5d, and 5f, 5h, 8d, 8g, and 8i which show data from multiple organoid lines.
Randomization	All organoid samples were analyzed equally and lines were used for experiments based on availability at the time. Therefore, no randomization was required.
Blinding	Although researchers were not blind to the organoid lines being used, they were blind to the age and sex of the individual from which the lines originated.

Reporting for specific materials, systems and methods

We require information from authors about some types of materials, experimental systems and methods used in many studies. Here, indicate whether each material, system or method listed is relevant to your study. If you are not sure if a list item applies to your research, read the appropriate section before selecting a response.

Materials & experimental systems

n/a	Involved in the study	
☐	☒	Antibodies
☐	☒	Eukaryotic cell lines
☒	☐	Palaeontology and archaeology
☒	☐	Animals and other organisms
☐	☒	Human research participants
☒	☐	Clinical data
☒	☐	Dual use research of concern

Methods

n/a	Involved in the study	
☒	☐	ChIP-seq
☐	☒	Flow cytometry
☒	☐	MRI-based neuroimaging

Antibodies

Antibodies used	Alexa Fluor 647-Conjugated Anti-Chromogranin A Antibody (1:100) Novus Biologicals NBP2-47850AF647<br>Alexa Fluor 647-Conjugated Mouse IgG2b kappa Isotype Control Antibody (1:100) Biolegend 400330<br>Anti-Chromogranin A Antibody (1:100) Agilent/Dako M086901-2

Anti-Chromogranin A Antibody (1:100) Millipore Sigma HPA017369-100UL
Anti-Cholecystokinin Antibody (1:100) Abcam Ab27441
Anti-Cytokeratin 20 Antibody (1:50) Thermo Fisher Scientific 17329-1-AP
Anti-GIP Antibody (1:100) Invitrogen PA5-76867
Anti-GLP-1 Antibody (1:100) Abcam Ab23468
Anti-Lysozyme Antibody (1:50) Novus Biologicals NBP100-63062
Anti-MUC2 Antibody (1:50) Novus Biologicals NBP1-31231
Anti-PYY Antibody (1:50) Mybiosource MBS9208739
Anti-Serotonin Antibody (1:100) Abcam ab66047
Anti-Somatostatin Antibody (1:100) R&D Systems mab2358
APC anti-human β2-microglobulin Antibody (1:25) Biolegend 316311
APC anti-human CD298 Antibody (1:25) Biolegend 341706
PE-conjugated Anti-Chromogranin antibody (1:100) BD Biosciences 564563
PE-conjugated Mouse IgG1 kappa Isotype Control Antibody (1:200) BD Biosciences 554680
TotalSeq™-B0251 anti-human Hashtag 1 Antibody (1:25) Biolegend 394631
TotalSeq™-B0252 anti-human Hashtag 2 Antibody (1:25) Biolegend 394633
TotalSeq™-B0253 anti-human Hashtag 3 Antibody (1:25) Biolegend 394635
TotalSeq™-B0254 anti-human Hashtag 4 Antibody (1:25) Biolegend 394637
TotalSeq™-B0255 anti-human Hashtag 5 Antibody (1:25) Biolegend 394639
TotalSeq™-B0256 anti-human Hashtag 6 Antibody (1:25) Biolegend 394641
TotalSeq™-B0257 anti-human Hashtag 7 Antibody (1:25) Biolegend 394643
TotalSeq™-B0258 anti-human Hashtag 8 Antibody (1:25) Biolegend 394645
TotalSeq™-B0259 anti-human Hashtag 9 Antibody (1:25) Biolegend 394647
Donkey anti-Goat IgG (H+L) Cross-Adsorbed Secondary Antibody, Alexa Fluor 488 (1:400) Invitrogen A-11055
Donkey anti-Mouse IgG (H+L) Cross-Adsorbed Secondary Antibody, Alexa Fluor 488 (1:400) Invitrogen A-21202
Donkey anti-Mouse IgG (H+L) Cross-Adsorbed Secondary Antibody, Alexa Fluor 647 (1:400) Invitrogen A-31571
Donkey anti-Rabbit IgG (H+L) Cross-Adsorbed Secondary Antibody, Alexa Fluor 647 (1:400) Invitrogen A-31573
Donkey anti-Rat IgG (H+L) Cross-Adsorbed Secondary Antibody, Alexa Fluor 488 (1:400) Invitrogen A-21208

Validation

Alexa Fluor 647-Conjugated Anti-Chromogranin A Antibody (Novus Biologicals NBP2-47850AF647): In this manuscript, rectoids that did not show CHGA staining also had very low numbers of CHGA+ cells using this antibody and rectoids that had high numbers of CHGA staining had elevated niumbers of CHGA+ cells using this antibody (please see Fig 8)

Alexa Fluor 647-Conjugated Mouse IgG2b kappa Isotype Control Antibody (Biolegend 400330): Please see company website (https://www.biolegend.com/en-us/products/alexa-fluor-647-mouse-igg2b-kappa-isotype-ctrl-2691)

Anti-ChgA antibody (Agilent/Dako M086901-2): In this manuscript, CHGA staining is not present in organoids that do not express CHGA mRNA, but is present in those that do express CHGA mRNA, at levels that match their RNA expression (please see Fig 4)

Anti-ChgA antibody (Millipore Sigma HPA017369-100UL): Please see company website (https://www.sigmaaldrich.com/catalog/product/sigma/hpa017369?lang=en&region=US)

Anti-Cytokeratin 20 antibody (Thermo Fisher Scientific 17329-1-AP: Please see company website (https://www.thermofisher.com/antibody/product/Cytokeratin-20-Antibody-Polyclonal/17329-1-AP)

Anti-GIP antibody (Invitrogen PA5-76867): Please see company website (https://www.thermofisher.com/antibody/product/GIP-Antibody-Polyclonal/PA5-76867)

Anti-Lysozyme antibody (Novus Biologicals NBP100-63062): Please see company website (https://www.novusbio.com/products/lysozyme-antibody-bgn-0696-5b1_nb100-63062)

Anti-MUC2 antibody (Novus Biologicals NBP1-31231): Please see company website (https://www.novusbio.com/products/muc2-antibody_nbp1-31231)

Anti-Serotonin antibody (Abcam ab66047): Please see company website (https://www.abcam.com/serotonin-antibody-ab66047.html)

Anti-Somatostatin antibody (R&D Systems mab2358): Please see company website (https://www.rndsystems.com/products/human-mouse-somatostatin-antibody-906552_mab2358)

Anti-GLP-1 Antibody (Abcam Ab23468): In this manuscript, GLP-1 staining is not present in rectoids that have low levels of GCG mRNA, but is present in those that reach levels of GCG mRNA consistent with normal rectal mucosa (please see Fig 8)

Anti-PYY Antibody (Mybiosource MBS9208739): Please see company website (https://www.mybiosource.com/polyclonal-human-antibody/pyy/9208739)

Anti-Cholecystokinin Antibody (Abcam Ab27441): Please see company website (https://www.abcam.com/cholecystokinin-antibody-ab27441.html)

PE-Conjugated Anti-ChgA antibody (BD Biosciences Cat# 564563): Please see company website (https://www.bdbiosciences.com/us/reagents/research/antibodies-buffers/cell-biology-reagents/cell-biology-antibodies/pe-mouse-anti-human-chromogranin-a-s21-537/p/564563)

PE-conjugated Mouse IgG1 kappa Isotype Control Antibody (BD Biosciences 554680): Please see company website (https://www.bdbiosciences.com/en-us/products/reagents/flow-cytometry-reagents/research-reagents/flow-cytometry-controls-and-lysates/pe-mouse-igg1-isotype-control.554680)

TotalSeq™-B0251 anti-human Hashtag 1 Antibody (Biolegend 394631)
TotalSeq™-B0252 anti-human Hashtag 2 Antibody (Biolegend 394633)
TotalSeq™-B0253 anti-human Hashtag 3 Antibody (Biolegend 394635)
TotalSeq™-B0254 anti-human Hashtag 4 Antibody (Biolegend 394637)
TotalSeq™-B0255 anti-human Hashtag 5 Antibody (Biolegend 394639)
TotalSeq™-B0256 anti-human Hashtag 6 Antibody (Biolegend 394641)
TotalSeq™-B0257 anti-human Hashtag 7 Antibody (Biolegend 394643)
TotalSeq™-B0258 anti-human Hashtag 8 Antibody (Biolegend 394645)
TotalSeq™-B0259 anti-human Hashtag 9 Antibody (Biolegend 394647): We validated all Hashtag antibodies using APC anti-human β2-microglobulin Antibody (Biolegend 316311) and APC anti-human CD298 Antibody (Biolegend 341706) to confirm binding efficiency as all Hashtag antibodies bind both β2-microglobulin and CD298. Please see company websites (https://www.biolegend.com/en-us/products/apc-anti-human-cd298-antibody-10327 and https://www.biolegend.com/en-ie/search-results/apc-anti-human-beta2-microglobulin-antibody-6910)

Eukaryotic cell lines

Policy information about cell lines

Cell line source(s)	(None of the lines below were obtained commercially)<br>L-WRN (Murine, Thad Stappenbeck)<br>HA-R-Spondin1-Fc 293T (Human, Calvin Kuo)<br>H357 (Human enteroid line, Harvard Digestive Disease Center)<br>H367 (Human rectoid line, Harvard Digestive Disease Center)<br>H368 (Human enteroid line, Harvard Digestive Disease Center)<br>H389 (Human enteroid line, Harvard Digestive Disease Center)<br>H393 (Human enteroid line, Harvard Digestive Disease Center)<br>H395 (Human enteroid line, Harvard Digestive Disease Center)<br>H407 (Human enteroid line, Harvard Digestive Disease Center)<br>H416 (Human enteroid line, Harvard Digestive Disease Center)<br>H439 (Human enteroid line, Harvard Digestive Disease Center)<br>H567 (Human rectoid line, Harvard Digestive Disease Center)<br>H587 (Human rectoid line, Harvard Digestive Disease Center)<br>H609 (Human rectoid line, Harvard Digestive Disease Center)<br>H616 (Human rectoid line, Harvard Digestive Disease Center)<br>H642 (Human rectoid line, Harvard Digestive Disease Center)<br>H645 (Human rectoid line, Harvard Digestive Disease Center)<br>H646 (Human rectoid line, Harvard Digestive Disease Center)
Authentication	None of the cell lines have been authenticated.
Mycoplasma contamination	All cell lines tested negative for mycoplasma.
Commonly misidentified lines<br>(See ICLAC register)	No commonly misidentified cell lines were used.

Human research participants

Policy information about studies involving human research participants

Population characteristics	All biopsies and resections were taken from phenotypically normal duodenal and rectal tissue of patients. Below are the age and sex characteristics of all tissues:<br><br>Age range for enteroid/rectoid lines: 12-21 (6 males, 10 females)<br>Age range duodenal mucosa RNA: 55-82 (1 male, 2 females)<br>Age range rectal mucosa RNA: 115-18 (3 females)<br><br>All lines marked for enteroid line use required esophagogastroduodenoscopy for various gastrointestinal complaints, but did not have any known gastrointestinal diagnoses at the time of the procedure. All lines marked for rectoid line use required colonoscopy for various gastrointestinal complaints, but did not have any known gastrointestinal diagnoses at the time of the procedure. Lines used for duodenal mucosa RNA were all diagnosed with pancreatic carcinoma requiring pancreaticoduodenectomy. Additional diagnoses are unknown to the researchers.
Recruitment	Subjects were approached by an IRB-trained research coordinator who asked if they had a few minutes to hear about a research study that they were eligible to participate in that day. Subjects were told that tissue samples would be stored in a Biobank and could be used to generate organoid lines and for genomic research. Subjects were informed that clinical information would be collected but stored in a HIPPA compliant way and that samples will always be deidentified with a unique study ID. Subjects were informed that participation in research is completely voluntary and that they could withdraw at any time. Subjects were given a chance to voice questions or concerns. There are no potential biases present.
Ethics oversight	Boston Children's Hospital IRB (P00000529)<br>Massachusetts General Hospital IRB (2003P001289)

Note that full information on the approval of the study protocol must also be provided in the manuscript.

Flow Cytometry

Plots

Confirm that:

- [x] The axis labels state the marker and fluorochrome used (e.g. CD4-FITC).
- [x] The axis scales are clearly visible. Include numbers along axes only for bottom left plot of group (a 'group' is an analysis of identical markers).
- [x] All plots are contour plots with outliers or pseudocolor plots.
- [x] A numerical value for number of cells or percentage (with statistics) is provided.

Methodology

Sample preparation	Organoids were incubated in Cell Recovery solution for 40-60 minutes at 4C to remove the Matrigel and then centrifuged at 500 x g for 5 minutes at 4°C. To achieve single cell suspension, organoids were then incubated in 500 µL of TrypLE Express at 37°C for 30 minutes and broken up by repeated pipetting using a bent P1000 pipette tip. Each sample was then diluted in 800µL of 20% fetal bovine serum (FBS) in Advanced DMEM/F12 and then centrifuged at 800 x g for 5 minutes at 4°C. To mark dead cells, each sample was then incubated in DAPI (1:1000) diluted in 2% FBS/2 mM EDTA/calcium-free DMEM for 20 minutes at room temperature, then centrifuged at 800 x g for 5 minutes at 4C and washed in 2% FBS/2 mM EDTA/DMEM. Cells were then incubated in 1% PFA for 15 minutes at room temperature, washed with 2% FBS/PBS and then permeabilized in 0.2% Tween 20 in 2% FBS/PBS for 15 minutes at 37°C. Following centrifugation, cells were resuspended in 0.1% Tween 20/2% FBS/2 mM EDTA in PBS with PE/Alexa Fluor 647-conjugated CHGA, PE-conjugated mouse IgG1, K isotype, Alexa Fluor 647-conjugated mouse IgG2b, K isotype, or with no antibody (the latter three acting as controls) for 30 minutes on ice. Cells were then washed in 0.1% Tween 20/2% FBS/2 mM EDTA in PBS, filtered through a 37-micron mesh, and then analyzed on a BD LSRFortessa flow cytometer using BD FACSDiva (v8.02) and FlowJo (v10.6.2).
Instrument	BD LSRFortessa
Software	BD FACSDiva v8.02 was used to collect flow cytometry data<br>FlowJo v10.6.2 was used to analyze flow cytometry data
Cell population abundance	CHGA positive cells ranged from 0.0-7.0% of the enteroid cell population, based on the exposure to different differentiation protocols. As cell were isolated from cultures growing only organoids, which we confirmed based on visual inspection, there was no need to further confirm purity of our samples.

Gating strategy

Organoid cells are differentiated from cellular debris based on their forward and side scatter area (FSC-A and SSC-A, respectively) parameters. Cells are then examined based on their FSC-A and FSC-Height (H) to exclude doublets. 4',6-diamidino-2-phenylindole (DAPI) staining is then utilized to identify dead cells, with DAPI high-positive cells being removed from futher gating. The CHGA-positive gate is set by using either PE-conjugated mouse IgG1, K isotype or Alexa Fluor 647-conjugated mouse IgG2b, K isotype.

☒ Tick this box to confirm that a figure exemplifying the gating strategy is provided in the Supplementary Information.
